# Supplementary material for: Field-induced quantum spin disordered state in spin-1/2 honeycomb magnet Na2Co2TeO6
Source: Nat Commun. 2021 Sep 21;12:5559. doi: 10.1038/s41467-021-25567-7 (PMC8455656; doi:10.1038/s41467-021-25567-7)
Supplement: Supplementary file 1 — Supplementary Information [file 41467_2021_25567_MOESM1_ESM.docx]

**Supplemental Material**

**Field-induced quantum spin disordered state in spin-1/2 honeycomb magnet Na_2_Co_2_TeO_6_**

Gaoting Lin^1^, Jaehong Jeong^2,3^, Chaebin Kim^2,4^, Yao Wang^5,6^, Qing Huang^7^, Takatsugu Masuda^8^, Shinichiro Asai^8^, Shinichi Itoh^9^, Gerrit Günther^10^, Margarita Russina^10^, Zhilun Lu^10,11^, Jieming Sheng^12,13,14^, Le Wang^14^, Jiucai Wang^15^, Guohua Wang^1^, Qingyong Ren^1,12,13^, Chuanying Xi^16^, Wei Tong^16^, Langsheng Ling^16^, Zhengxin Liu^15^, Liusuo Wu^14^, Jiawei Mei^14^, Zhe Qu^16,17^, Haidong Zhou^7^, Xiaoqun Wang^1^, Je-Geun Park^2,4^, Yuan Wan^5,6,18^, *, and Jie Ma^1^, *

^1^*Key Laboratory of Artificial Structures and Quantum Control (Ministry of Education), Shenyang National Laboratory for Materials Science, School of Physics and Astronomy, Shanghai Jiao Tong University, Shanghai 200240, China*

^2^*Department of Physics and Astronomy, Seoul National University, Seoul 08826, Republic of Korea*

^3^*Center for Correlated Electron Sciences, Institute for Basic Science (IBS), Seoul 08826, Republic of Korea*

^4^*Center for Quantum Materials, Seoul National University, Seoul 08826, Republic of Korea*

^5^*Institute of Physics, Chinese Academy of Sciences, Beijing 100190, China*

^6^*University of Chinese Academy of Sciences, Beijing 100049, China*

^7^*Department of Physics and Astronomy, University of Tennessee, Knoxville, Tennessee 37996, USA*

^8^*Institute for Solid State Physics, University of Tokyo, Kashiwanoha, Kashiwa, Chiba 277-8581, Japan*

^9^*Institute of Materials Structure Science, High Energy Accelerator Research Organization, Tsukuba 305-0801, Japan*

^10^*Helmholtz-Zentrum Berlin für Materialien und Energie, Hahn-Meitner-Platz 1, Berlin 14109, Germany*

^11^*The Henry Royce Institute and Department of Materials Science and Engineering, The University of Sheffield, Sir Robert Hadfield Building, Sheffield, S1 3JD, United Kingdom*

^12^*Spallation Neutron Source Science Center, Dongguan 523803, China*

^13^*Institute of High Energy Physics, Chinese Academy of Sciences, Beijing 100049, China*

^14^*Shenzhen Institute for Quantum Science and Engineering (SIQSE) and Department of Physics, Southern University of Science and Technology (SUSTech), Shenzhen, Guangdong 518055, China*

^15^*Department of Physics, Renmin University of China, Beijing 100872, China*

^16^*Anhui Province Key Laboratory of Condensed Matter Physics at Extreme Conditions, High Magnetic Field Laboratory, Hefei Institutes of Physical Sciences, Chinese Academy of Science, Hefei, Anhui 230031, China*

^17^*CAS Key Laboratory of Photovoltaic and Energy Conservation Materials, Hefei Institutes of Physical Sciences, Chinese Academy of Sciences, Hefei, Anhui 230031, China*

^18^*Songshan Lake Materials Laboratory, Dongguan, Guangdong 523808, China*

*Corresponding author：[yuan.wan@iphy.ac.cn](mailto:yuan.wan@iphy.ac.cn)

[jma3@sjtu.edu.cn](mailto:jma3@sjtu.edu.cn)

1. **Crystal structure**

**
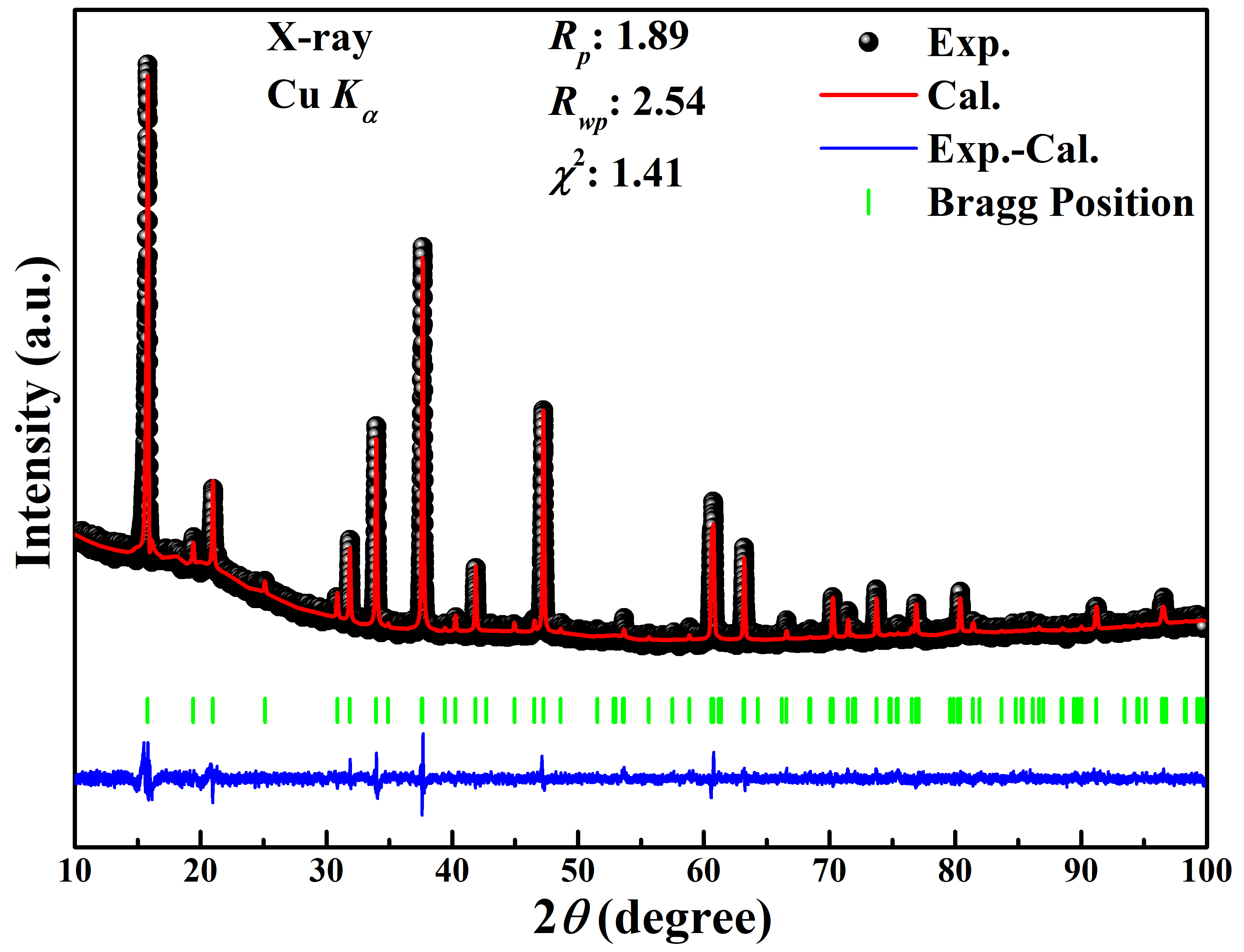
**

**Supplementary Figure 1: Powder X-ray diffraction of Na_2_Co_2_TeO_6_.** The powder x-ray diffraction pattern was recorded using Cu *K_α_* radiation at room temperature. The difference between experimental and calculated patterns is shown by the blue solid lines. The vertical bars indicate the positions of allowed Bragg peaks.

The measured diffraction patterns were analyzed by using the Rietveld refinement technique (by employing the FULLPROF computer program). The Rietveld analysis reveals that the compound crystallizes in the hexagonal symmetry with space group P6322 (No. 182).

**2. Heat capacity**


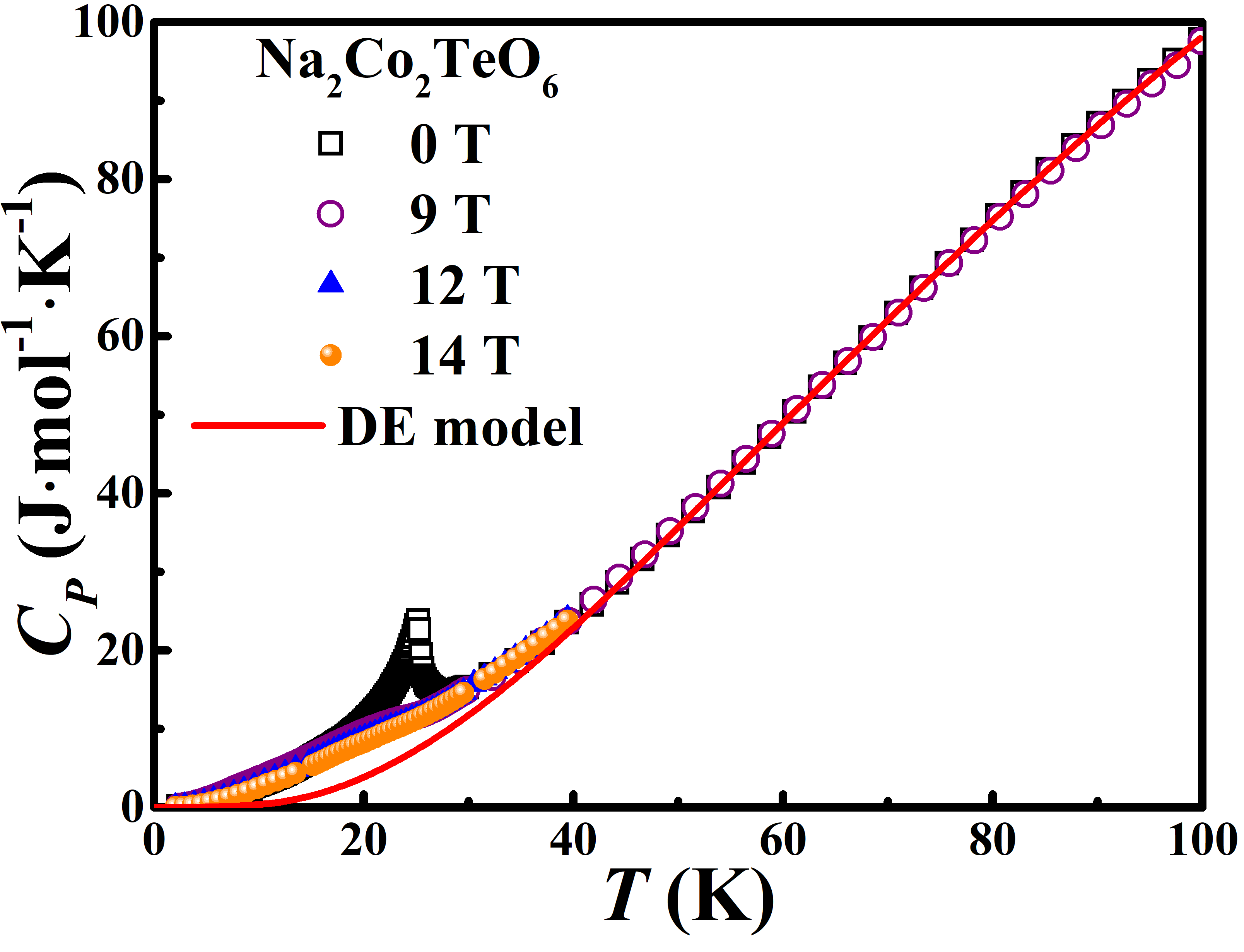


**Supplementary Figure 2:** **Heat capacity of Na_2_Co_2_TeO_6_ under different field.** Heat capacity *C_P_* as a function of *T* for NCTO. The red line is the calculated *C_V_*(*T*) using Eq. S(1).

Since NCTO is a Mott insulator [1,2], the electronic contribution to the heat capacity is not considered. Hence, for a reasonable description of lattice heat capacity *C_V_*(*T*), a combined Debye-Einstein (DE) model should be employed, where *C_V_*(*T*) is defined by the following expression [3]:

$C_{\text{V}}(T)=9R\frac{\sum_{i=1}^{3} a_{i}}{n-1}x_{D}^{-3}\int_{0}^{x_{D}} \frac{x^{4}e^{x}}{\left(e^{x}-1\right)^{2}}dx+3R\sum_{i=1}^{3} \left[a_{_{i}}\frac{x_{E_{i}}^{2}e^{x_{E_{i}}}}{\left(e^{x_{E_{i}}}-1\right)^{2}}\right]$, S(1)

where *x_D_* and *x_E_* are defined via *x_D_* = *Θ_D_*/*T* and *x_E_* = *Θ_E_*/*T*, *Θ_D_* and *Θ_E_* are the Debye and Einstein temperatures, respectively. The parameter *n* is the number of atoms per formula unit, and *R* is the molar gas constant. The constants *a_i_* are adjust parameters. The first term in Eq. S(1) represents the Debye heat capacity related to the acoustic modes, and the second term is a sum of Einstein phonon contributions related to optic modes. The red curve shows the fitted *C_P_*(*T*) by Eq. S(1) over the temperature range from about 2 to 100 K in Supplementary Fig. 1 using the *Θ_D_* = 160.2 K, $\text{Θ}_{\text{E}_{1}}$= 113.9 K, $\text{Θ}_{\text{E}_{2}}$= 234.6 K, $\text{Θ}_{\text{E}_{3}}$= 467.8 K. Hence, we can extract the magnetic contribution *C*_mag_(*T*) from the measured *C_P_*(*T*), *C*_mag_(*T*) = *C_P_*(*T*) - n*C_V_*(*T*).

**3. High-field electron spin resonance**

The EPR spectra are well ﬁtted by Dysonian line shape given by:

$\frac{dP}{dB}\propto\frac{d}{dB}\left[\frac{2\Delta B+4a\left(B-B_{r}\right)}{\Delta B^{2}+4\left(B-B_{r}\right)^{2}}+\frac{2\Delta B+4a\left(B+B_{r}\right)}{\Delta B^{2}+4\left(B+B_{r}\right)^{2}}\right]$. S(2)

This is an asymmetric Lorentzian line, which includes absorption and dispersion, denoting the dispersion-to-absorption (D/A) ratio. *B_r_* and *ΔB* are the resonance field and full-linewidth at half-maximum, respectively. Compared to the fitted results of 26 K, higher resonance fields at 50 K indicate the presence of short-range magnetic correlations above *T*_N_ (Supplementary Fig. 3).

**
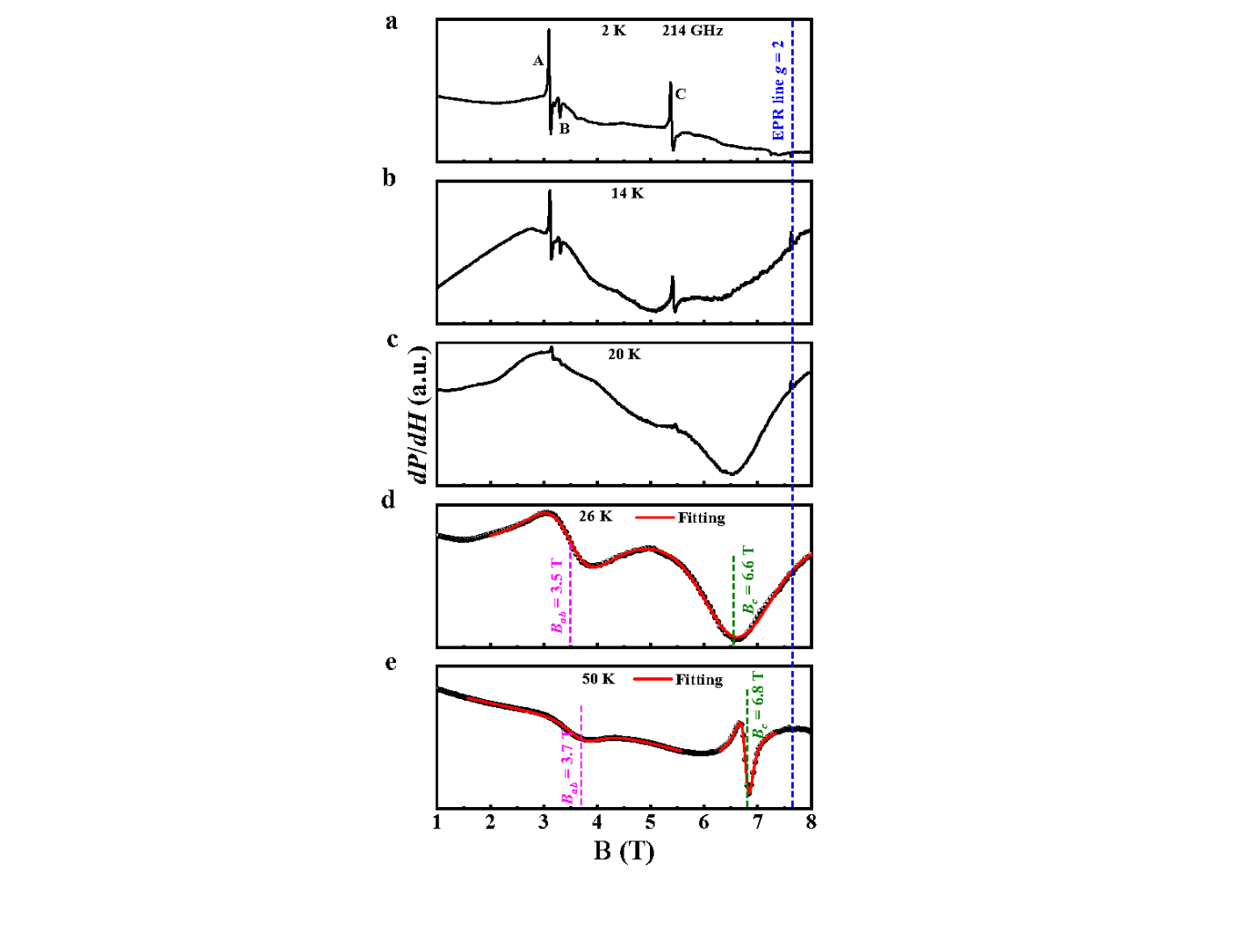
**

**Supplementary Figure 3: ESR spectra of Na_2_Co_2_TeO_6_ at different temperatures.** ESR spectra with the frequency *v* = 214 GHz at different temperatures. A weak but observable EPR signal marked with a blue dashed line indicates PM impurities in our sample, not affected by our results. The red lines are the fitted EPR lines using Eq. S(2).

**4. Linear spin-wave theory for zigzag ground states**

**4.1 Symmetries and model**

The space group of NCTO is P6_3_22 (No.182). This material can be naturally viewed as a magnetic bilayer with AB stacking. Co^2+^ ions at crystallographic positions (0, 0, 1/4) (Wyckoff position 2*b*) and (2/3, 1/3, 1/4) (Wyckoff position 2*d*) form the first layer (Supplementary Fig. 4a). The system possesses a 3-fold rotational symmetry at these sites with respect to the crystallographic *c* axis and 2-fold rotational symmetry with respect to the three-neighboring links. Co^2+^ at (0, 0, 3/4) (Wyckoff position 2*b*) and (1/3, 2/3, 3/4) (Wyckoff position 2*d*) form the second layer. The symmetry properties of the second layer are identical to the first.

To understand the dynamical spin-spin correlations, we compare the INS data to the solution of H-K Hamiltonian using conventional LSWT for zigzag order. We propose a phenomenological, symmetry-constrained pseudo-spin *J*_eff_ = 1/2 exchange Hamiltonian for this material. We neglect the interlayer coupling as the previous analysis seems to suggest it to be weak. Consider a pair of nearest neighbors *ij*, where *i* ∈ A and *j* ∈ B. The 2-fold rotational symmetry with respect to the bond *ij* allows the following exchange interactions:

$H_{ij}=J_{1}\mathbf{S}_{i}\cdot\mathbf{S}_{j}+IS_{i}^{c}S_{j}^{c}+J_{K}(\mathbf{S}_{i}\cdot\mathop{n}\limits^{\wedge} _{ij})(\mathbf{S}_{j}\cdot\mathop{n}\limits^{\wedge} _{ij})+(J_{\Gamma}+J_{D})(\mathbf{S}_{i}\cdot\varepsilon^{\wedge} _{ij})S_{j}^{c}+(J_{\Gamma}-J_{D})(\mathbf{S}_{j}\cdot\varepsilon^{\wedge} _{ij})S_{i}^{c}$ S(3)

Here$S_{i}^{c}$is the projection of the spin along the crystallographic *c* axis.$\mathop{n}\limits^{\wedge} _{ij}$is the unit vector pointing from site *i* to *j*, $\varepsilon^{\wedge} _{ij}\equiv\mathop{c}\limits^{\wedge} \times\mathop{n}\limits^{\wedge} _{ij}$. Therefore,$\{\mathop{n}\limits^{\wedge} _{ij},\varepsilon^{\wedge} _{ij},\mathop{c}\limits^{\wedge} \}$form a (link dependent) right-hand frame (Supplementary Fig. 4b). The 2-fold rotational symmetry with respect to$\mathop{n}\limits^{\wedge} _{ij}$forbids the coupling between the spin component parallel with the$\mathop{n}\limits^{\wedge} _{ij}$axis and the components orthogonal to it. This eliminates 4 coupling constants. 3 × 3 - 4 = 5 coupling constants remain.

We recognize *I* as the Ising exchange interaction, *J_K_* as the pseudo-dipolar interaction, *J_D_* the DM (Dzyaloshinskii–Moriya) interaction, and finally, *J_Γ_* the symmetric off-diagonal interaction. Crucially, the pseudo-dipolar interaction is not the Kitaev interaction proposed by J. Chaloupka et al. [4] in that the said Kitaev interaction uses a spin coordinate system different from here. However, it is possible to express the said Kitaev interaction in terms of these coupling constants. We also would like to include the second and third neighbor exchange interactions. Liu and Khaliullin [1] argue that the further neighbor exchange interactions tend to be more isotropic as the spin anisotropy resulted from different superexchange paths average out.

We thus take these further neighbor exchange interactions to be of Heisenberg type. Hence, we write the Hamiltonian as *H* = *H*^(I)^ + *H*^(II)^, where *H*^(I)^ and *H*^(II)^ respectively describe the first and second layer:

$H^{(k)}=\sum_{\left\langle ij\right\rangle\in k} H_{ij}+J_{2}\sum_{\left\langle\left\langle ij\right\rangle\right\rangle\in k} \mathbf{S}_{i}\cdot\mathbf{S}_{j}+J_{3}\sum_{\left\langle\left\langle\left\langle ij\right\rangle\right\rangle\right\rangle\in k} \mathbf{S}_{i}\cdot\mathbf{S}_{j}.$ (*k* = I, II). S(4)


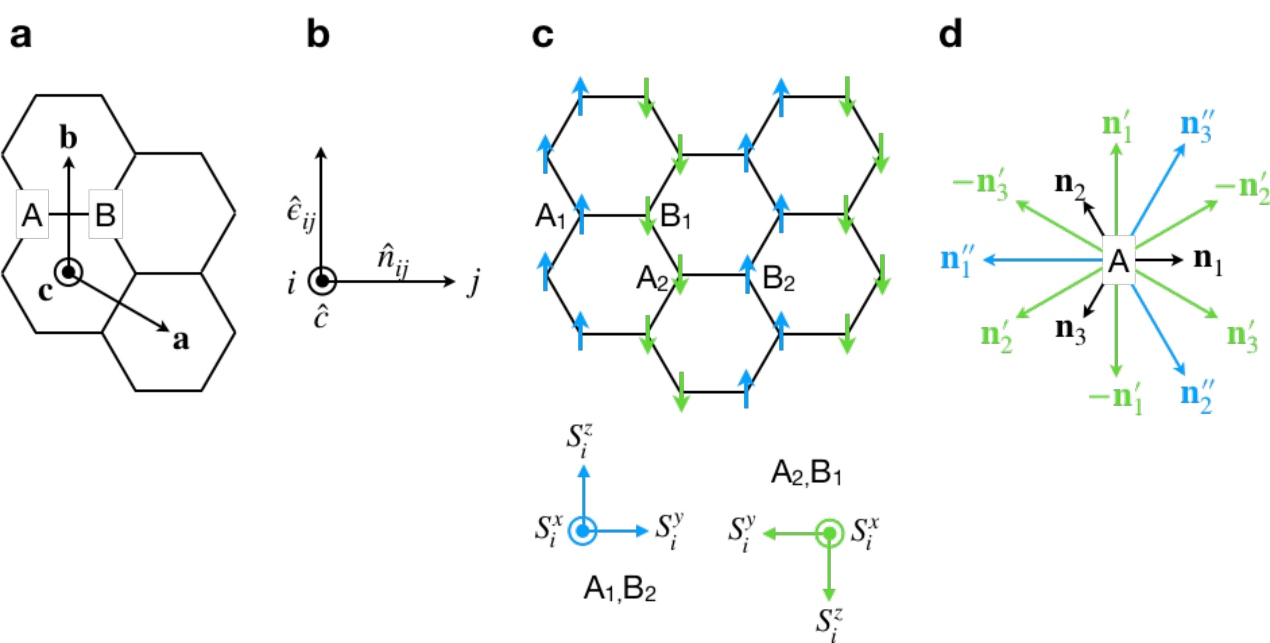


**Supplementary Figure 4: Structure of Na_2_Co_2_TeO_6_.** **a** A single layer of the honeycomb lattice. *a*, *b*, *c* is the crystallographic primitive vectors. A and B label the two crystallographic sublattices. **b** The right-hand frame is associated with a link *ij*. **c** Zigzag ground state. A_1,2_ and B_1,2_ label the magnetic sublattices. The local spin frames are also shown. **d** The nearest, second, and third neighbors of an A sublattice site and the associated vectors.

The first, second, and third summations are, respectively, over nearest, second, and third neighbors in the same layer (Supplementary Fig. 4d). However, since it has been shown experimentally that the inter-layer spin correlations are short-ranged, we shall treat the layers as independent and perform all the calculations in the effective single-layer model.

**4.2 Constraining model parameters**

The experiment has shown that NCTO shows zigzag order in its ground state. Refinement suggests that the magnetic moments are along the crystallographic *b* axis with a small *c* component. As a first approximation, we may take the moments to be in the basal plane. This implies that *J_Γ_* and *J_D_* must be approximately zero.

One of the symmetry-equivalent zigzag orders is shown in Supplementary Fig. 4c. There are four magnetic sublattices, labeled A_1,2_ and B_1,2_. We compute the Weiss molecular field on each spin. We find the molecular fields due to the *J_Γ_* and *J_D_* terms are all along the crystallographic *c* axis:

$h_{i}^{c}=\left\{\begin{matrix} 2\left(J_{\Gamma}-J_{D}\right)S \\ -2\left(J_{\Gamma}-J_{D}\right)S \\ -2\left(J_{\Gamma}+J_{D}\right)S \\ 2\left(J_{\Gamma}+J_{D}\right)S \end{matrix}\right.\begin{matrix} \left(i\in A_{1}\right) \\ \left(i\in A_{2}\right) \\ \left(i\in B_{1}\right) \\ \left(i\in B_{2}\right) \end{matrix}$ S(5)

For the magnetic moment to be in the basal plane, the molecular fields along the crystallographic *c* axis must vanish. This implies *J_Γ_* = *J_D_* = 0.

The independent parameters are thus *J*_1,2,3_, *J_K_*, and *I*. The previous analysis has shown that the *J*_1_-*J*_2_-*J*_3_ model may stabilize the zigzag type magnetic order. However, *J*_1,2,3_ alone, being isotropic in spin space, cannot force the magnetic moments to be in any particular direction ― this is achieved by *J_K_* and *I*. We now determine the sign of *J_K_* and *I*. To this end, we consider the following trial state: the spins form a collinear zigzag order similar to Supplementary Fig. 4c. The magnetic moment takes a direction$\mathop{m}\limits^{\wedge}$that is not necessarily along the crystallographic *b* axis. The energy per unit cell is given by:

$\frac{E(\mathop{m}\limits^{\wedge} )}{N_{cell}}=-\frac{J_{K}}{2}(m^{a*})^{2}+\frac{3J_{K}}{2}(m^{b})^{2}+I(m^{c})^{2}.$ S(6)

Here, *m^a^*^*^, *m^b^,* and *m^c^* are the projection of$\mathop{m}\limits^{\wedge}$along the *a**, *b*, and *c* axis. For the moment to be in the *b* axis, we must have:

$J_{K}<0, I>-\frac{3\left|J_{K}\right|}{2}.$ S(7)

**4.3 Spin wave calculations**

We now compute the spin-wave spectrum. For the sake of simplicity, we set *J*_2_ = *I* = 0. To this end, we orient the local spin frames such that the local$S_{i}^{z}$axis coincides with the magnetic moment in the ground state. The specific choices are given in Supplementary Fig. 4c. We then employ the Holstein-Primakoff transformation to the leading order in 1/*S*:

$S_{r}^{x}=\sqrt{S}x_{r}; S_{r}^{y}=\sqrt{S}y_{r}; S_{r}^{z}=S-\frac{x_{r}^{2}+y_{r}^{2}}{2}.$ S(8)

where *x_r_* and *y_r_* obey the commutation relation: [*x_r_*, *y_r_*] = *i*. Substituting it into the Hamiltonian *H*^(I)^ and expand to the second-order,

$\begin{matrix} H^{(\text{I})}=\frac{-J_{1}S+3J_{3}S-3J_{K}S/2}{2}\sum_{r} (x_{r}^{2}+y_{r}^{2})+J_{1}S\sum_{r\in A} \sum_{i} x_{r}x_{r+n_{i}}+J_{3}S\sum_{r\in A} \sum_{i} x_{r}x_{r+n_{i}^{''}} \\ -\sum_{r\in A} (J_{1}+J_{K})y_{r}y_{r+n_{1}}+(J_{1}+\frac{J_{K}}{4})y_{r}y_{r+n_{2}}+(J_{1}+\frac{J_{K}}{4})y_{r}y_{r+n_{3}}-J_{3}S\sum_{r\in A} \sum_{i} y_{r}y_{r+n_{i}^{''}}. \end{matrix}$ S(9)

Here $\mathbf{n}_{1,2,3},\pm\mathbf{n}_{1,2,3}^{'},\text{and }\mathbf{n}_{1,2,3}^{''}$are vectors associated with the nearest, second, and third neighbors. Their definitions are given in Supplementary Fig. 4d.

We note that Hamiltonian's translation symmetry is the same as the lattice (i.e., higher than the translation symmetry of the magnetic ground state). Thus, we may block diagonalize the Hamiltonian by switching to the momentum space:

$x_{r}=\frac{1}{\sqrt{N_{cell}}}\sum_{\mathbf{q}\in1\text{BZ}} x_{A/B}(\mathbf{q})\exp(i\mathbf{q}\cdot\mathbf{r}), y_{r}=\frac{1}{\sqrt{N_{cell}}}\sum_{\mathbf{q}\in1\text{BZ}} y_{A/B}(\mathbf{q})\exp(i\mathbf{q}\cdot\mathbf{r}).$ S(10)

where the summation is over the first BZ (rather than the magnetic BZ). The commutation relation is: [*x_α_*(**q**), *y_α_*(-**q**)] = *i*. The Hamiltonian now reads,

$\begin{matrix} H^{(\text{I})}=\frac{1}{2}\sum_{\mathbf{q}} x^{\dagger}(\mathbf{q})X(\mathbf{q})x(\mathbf{q})+y^{\dagger}(\mathbf{q})Y(\mathbf{q})y(\mathbf{q}) \\ =\frac{1}{2}\sum_{\mathbf{k}} \left(x_{A}(-\mathbf{q}),x_{B}(-\mathbf{q})\right)\left(\begin{matrix} X_{AA}(\mathbf{q}) & X_{AB}(\mathbf{q}) \\ X_{AB}^{*}(\mathbf{q}) & X_{AA}(\mathbf{q}) \end{matrix}\right)\left(\begin{matrix} x_{A}(\mathbf{k}) \\ x_{B}(\mathbf{k}) \end{matrix}\right) \\ +\left(y_{A}(-\mathbf{q}),y_{B}(-\mathbf{q})\right)\left(\begin{matrix} Y_{AA}(\mathbf{q}) & Y_{AB}(\mathbf{q}) \\ Y_{AB}^{*}(\mathbf{q}) & Y_{AA}(\mathbf{q}) \end{matrix}\right)\left(\begin{matrix} y_{A}(\mathbf{k}) \\ y_{B}(\mathbf{k}) \end{matrix}\right), \end{matrix}$ S(11)

where

$$\begin{matrix} X_{AA}(\mathbf{q})/S=-J_{1}-3J_{K}/2+3J_{3}; \\ X_{AB}(\mathbf{q})/S=J_{1}\sum_{i} e^{i\mathbf{q}\cdot\mathbf{n}_{i}}+J_{3}\sum_{i} e^{i\mathbf{q}\cdot\mathbf{n}_{i}^{''}}; \\ Y_{AA}(\mathbf{q})/S=-J_{1}-3J_{K}/2+3J_{3}; \\ Y_{AB}(\mathbf{q})/S=-(J_{1}+J_{K})e^{i\mathbf{q}\cdot\mathbf{n}_{1}}+(J_{1}+J_{K}/4)\sum_{i=2,3} e^{i\mathbf{q}\cdot\mathbf{n}_{i}}-J_{3}\sum_{i} e^{i\mathbf{q}\cdot\mathbf{n}_{i}^{''}}. \end{matrix}$$

Note *X*(**q**) and *Y* (**q**) must be positive semi-definite for all **q**.

We are now ready to diagonalize the Hamiltonian *H*^(I)^. To this end, we perform the SVD:

*X*(**q**)^1/2^*Y*(**q**)^1/2^ = *U*(**q**)Ω(**q**)*V*^†^(**q**). ⇒ *Y*(**q**)^1/2^*X*(**q**)^1/2^ = *V*(**q**)Ω(**q**)*U*(**q**)^†^. S(12)

Ω(**q**) = diag(*ω*_1_(**q**), *ω*_2_(**q**)). The second equation follows from the first by the hermiticity of *X*(**q**) and *Y*(**q**). Furthermore,

*X*(**q**)* = *X*(-**q**), *Y*(**q**)* = *Y*(-**q**). ⇒ *U*(**q**)* = *U*(-**q**), *V*(**q**)* = *V*(-**q**), Ω(**q**)* = Ω(-**q**). S(13)

The latter relations may be used as a gauge-fixing condition on *U*(**q**), *V*(**q**). We define a new set of variables:

$\begin{matrix} x_{\alpha}(\mathbf{q})=\sum_{\lambda} \left[(Y(\mathbf{q}))^{1/2}V(\mathbf{q})\right]_{\alpha\lambda}\frac{b_{\lambda}(\mathbf{q})+b_{\lambda}^{\dagger}(-\mathbf{q})}{\sqrt{2\omega_{\lambda}(\mathbf{q})}}; \\ y_{\alpha}(\mathbf{q})=\sum_{\lambda} \left[(X(\mathbf{q}))^{1/2}U(\mathbf{q})\right]_{\alpha\lambda}\frac{b_{\lambda}(\mathbf{q})-b_{\lambda}^{\dagger}(-\mathbf{q})}{i\sqrt{2\omega_{\lambda}(\mathbf{q})}}. \end{matrix}$ S(14)

Here, *α* = A, B. *λ* = 1, 2 labels the two spin wave branches. *b_λ_*(**k**) annihilates a magnon with label *λ* and momentum **k**. *ω_λ_*(**k**) is the dispersion relation of the spin-wave. It’s straightforward to show that the above transformation preserves the commutation relations and constitutes a canonical transformation. Substituting the above into *H*^(I)^,

$H^{(\text{I})}=\sum_{\mathbf{k}\lambda} \omega_{\lambda}(\mathbf{k})\left(b_{\lambda}^{\dagger}(\mathbf{k})b_{\lambda}(\mathbf{k})+1/2\right)$ S(15)

As *H*^(II)^ is isomorphic to *H*^(I)^, it is diagonalized in the same vein.

**4.4 Dynamic spin structure factor**

We now compute the dynamic spin structure factor S*^αβ^*(**q**, *ω*). We first relate the Fourier transform of the magnetic moment to *x*_A,B_(**q**) and *y*_A,B_(**q**):

$\mu^{a*}(\mathbf{q})=\sqrt{S}[y_{A}(\mathbf{q}+\mathbf{G}_{1}/2)-e^{i\frac{2\pi}{3}}y_{B}(\mathbf{q}+\mathbf{G}_{1}/2)]; \mu^{c}(\mathbf{q})=\sqrt{S}[x_{A}(\mathbf{q})+x_{B}(\mathbf{q})].$ S(16)

We have omitted the Bohr magneton, etc. Note *â**, $\hat{b}$, *ĉ* form an orthogonal frame (the crystallographic *â*, $\hat{b}$, *ĉ* do not form orthogonal frame). The *b* direction is the ordering direction of the magnetic moment; scattering in this direction only produces elastic component within the quadratic approximation and shall be omitted. **G**_1_/2 is the ordering wave vector.

The additional phase factor exp($-i\frac{2\pi}{3}$) requires explanation. The boost vector **G**_1_/2 accounts for the staggering of the local *S^y^* axis. However, it introduces an unwanted phase difference -(**n**_1_∙**G**_1_)/2 = -2*π*/3 between the A and B sub-lattices. The factor mentioned above cancels this phase difference:

$\begin{matrix} \mu^{a*}(\mathbf{q})\approx\sum_{r\in A_{1}} y_{r}e^{-i\mathbf{q}\cdot\mathbf{r}}-\sum_{r\in A_{2}} y_{r}e^{-i\mathbf{q}\cdot\mathbf{r}}-\sum_{r\in B_{1}} y_{r}e^{-i\mathbf{q}\cdot\mathbf{r}}-\sum_{r\in B_{2}} y_{r}e^{-i\mathbf{q}\cdot\mathbf{r}} \\ =\sum_{r\in A_{1}} y_{r}e^{-i(\mathbf{q}+\mathbf{G}_{1}/2)\cdot\mathbf{r}}+\sum_{r\in A_{2}} y_{r}e^{-i(\mathbf{q}+\mathbf{G}_{1}/2)\cdot\mathbf{r}}-\sum_{r\in B_{1}} y_{r}e^{-i(\mathbf{q}+\mathbf{G}_{1}/2)\cdot(\mathbf{r}-\mathbf{n}_{1})}-\sum_{r\in B_{2}} y_{r}e^{-i(\mathbf{q}+\mathbf{G}_{1}/2)\cdot(\mathbf{r}-\mathbf{n}_{1})} \\ =y_{A}(\mathbf{q}+\mathbf{G}_{1}/2)-e^{i\frac{2\pi}{3}}y_{B}(\mathbf{q}+\mathbf{G}_{1}/2). \end{matrix}$ S(17)

We thus find the dynamic spin structure factors at zero temperature:

$\begin{matrix} S^{a*a*}(\mathbf{q},\omega)=S\sum_{\lambda} Z_{\lambda}^{a*}(\mathbf{q}+\mathbf{G}_{1}/2)\delta\left(\omega-\omega_{\lambda}(\mathbf{q}+\mathbf{G}_{1}/2)\right). \\ S^{cc}(\mathbf{q},\omega)=S\sum_{\lambda} Z_{\lambda}^{c}(\mathbf{q})\delta\left(\omega-\omega_{\lambda}(\mathbf{q})\right). \end{matrix}$ S(18)

The cross-correlations vanish due to the momentum mismatch. The spectral weights$Z_{\lambda}^{a*}(\mathbf{q})$and$Z_{\lambda}^{c}(\mathbf{q})$are given by

$\begin{matrix} Z_{\lambda}^{a*}(\mathbf{q})=\frac{1}{2\omega_{\lambda}(\mathbf{q})}\left[U(\mathbf{q})^{\dagger}X(\mathbf{q})^{1/2}\left(\begin{matrix} 1 & -e^{i\frac{2\pi}{3}} \\ -e^{-i\frac{2\pi}{3}} & 1 \end{matrix}\right)X(\mathbf{q})^{1/2}U(\mathbf{q})\right]_{\lambda\lambda}. \\ Z_{\lambda}^{c}(\mathbf{q})=\frac{1}{2\omega_{\lambda}(\mathbf{q})}\left[V(\mathbf{q})^{\dagger}Y(\mathbf{q})^{1/2}\left(\begin{matrix} 1 & 1 \\ 1 & 1 \end{matrix}\right)Y(\mathbf{q})^{1/2}V(\mathbf{q})\right]_{\lambda\lambda}. \end{matrix}$ S(19)

We are now ready to compute the powder average. We first note that the nominal bilayer system is equivalent to a single layer system as we have assumed no correlation between the two layers. Furthermore, *S^αβ^*(**q**, *ω*) only depends on the in-plane component. Let **q** = *q*(cos*ϕ*sin*θ*, sin*ϕ*sin*θ*, cos*θ*). Here, the three components are expressed in those mentioned above *a*bc* orthogonal frame. The cross-section is given by:

S(20)

The pre-factors are due to the projection operator (i.e., only components transverse to **q** contribute). The power-average is given by:

S(21)

**4.5 Results for typical parameters**


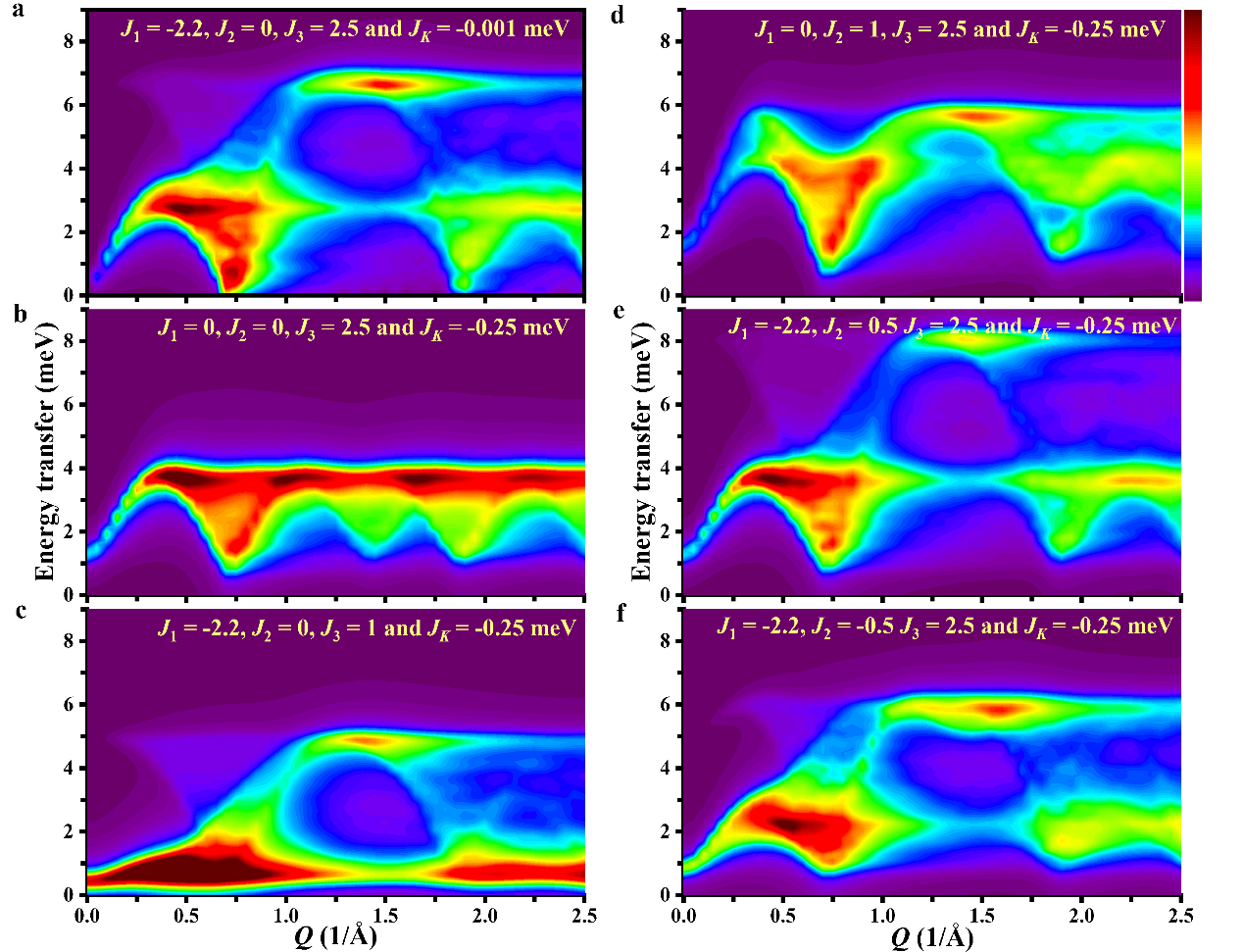


**Supplementary Figure 5: Calculated INS spectra.** LSWT calculations and corresponding powder averages with different parameters.


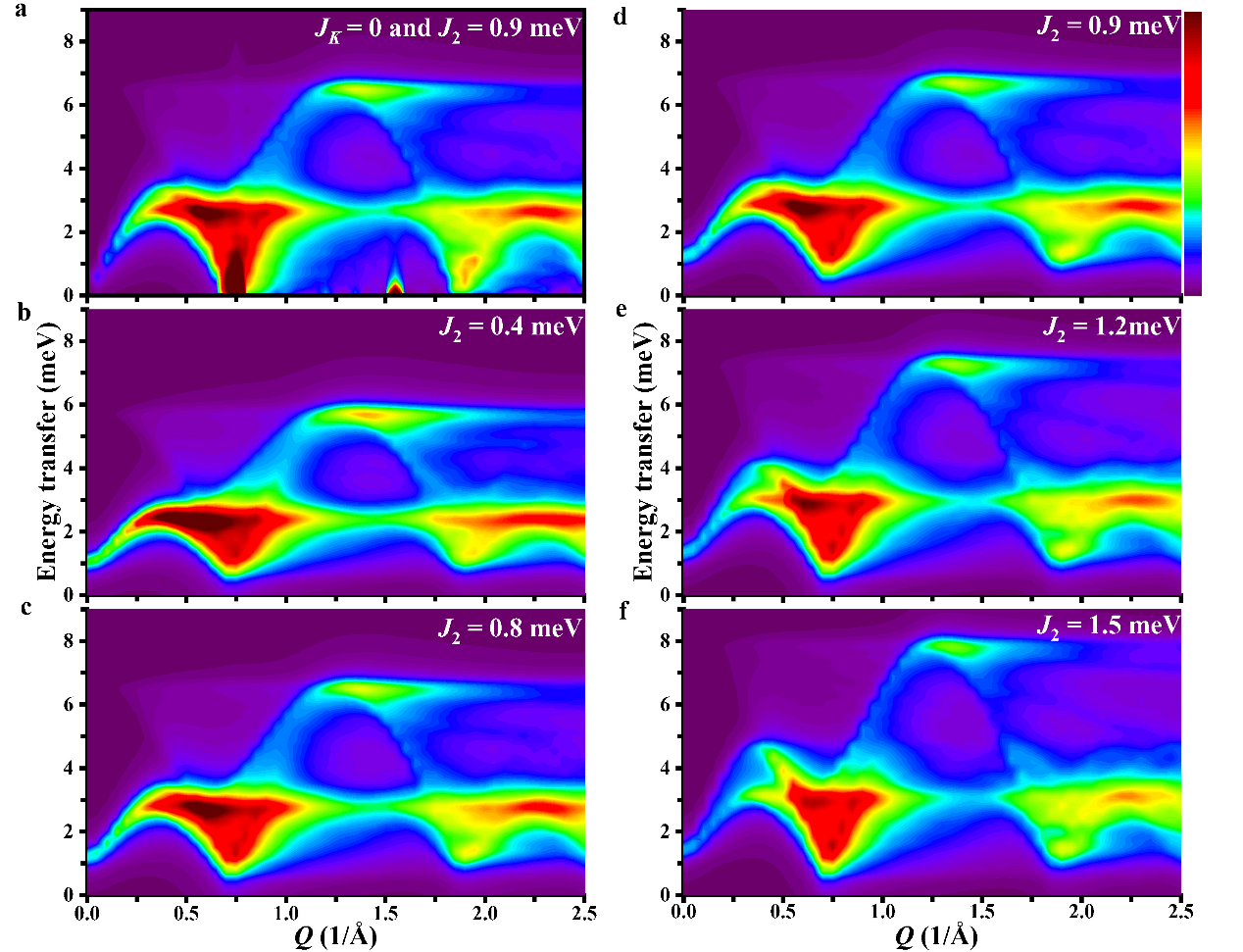


**Supplementary Figure 6: Calculated INS spectra.** LSWT calculations and corresponding powder averages. **a** The calculated spectrum for the parameters corresponding to (*J*_1_, *J*_2_, *J*_3_, *J_K_*) = (-1.6, 0.9, 1.6, 0) meV with a zigzag ground state. **b-f** The calculated spectrum for the constant parameters (*J*_1_, *J*_3_, *J_K_*) = (-1.6, 1.6, -0.25) meV with different *J*_2_.

**4.6 Transformation into the general form**

The Hamiltonian connected with the spin at position$\mathbf{S}_{\mathbf{r}_{i}}$reads,

$\begin{matrix} H_{i}=J_{1}\sum_{k=1,2,3} \mathbf{S}_{\mathbf{r}_{i}}\cdot\mathbf{S}_{\mathbf{r}_{i}+\mathbf{n}_{k}}+I(\mathbf{S}_{\mathbf{r}_{i}}\cdot\hat{c})(\mathbf{S}_{\mathbf{r}_{i}+\mathbf{n}_{k}}\cdot\hat{c})+J_{K}(\mathbf{S}_{\mathbf{r}_{i}}\cdot\mathbf{n}_{k})(\mathbf{S}_{\mathbf{r}_{i}+\mathbf{n}_{k}}\cdot\mathbf{n}_{k}) \\ \text{ }+\sum_{k=1,2,3} (J_{\Gamma}+J_{D})(\mathbf{S}_{\mathbf{r}_{i}}\cdot\hat{\varepsilon}_{k})(\mathbf{S}_{\mathbf{r}_{i}+\mathbf{n}_{k}}\cdot\hat{c})+(J_{\Gamma}-J_{D})(\mathbf{S}_{\mathbf{r}_{i}+\mathbf{n}_{k}}\cdot\hat{\varepsilon}_{k})(\mathbf{S}_{\mathbf{r}_{i}}\cdot\hat{c}) \end{matrix}$ S(22)

Express the constant vector directly,

$\begin{matrix} \hat{\mathbf{n}}_{\gamma_{1}}=\frac{1}{\sqrt{2}}(\hat{i}-\hat{j}),\text{ }\hat{\mathbf{n}}_{\gamma_{2}}=\frac{1}{\sqrt{2}}(\hat{j}-\hat{k}),\text{ }\hat{\mathbf{n}}_{\gamma3}=\frac{1}{\sqrt{2}}(\hat{k}-\hat{i}), \\ \hat{\varepsilon}_{i,i+\gamma_{1}}=\frac{1}{\sqrt{6}}(\hat{i}+\hat{j}-2\hat{k}),\text{ }\hat{\varepsilon}_{i,i+\gamma_{2}}=\frac{1}{\sqrt{6}}(-2\hat{i}+\hat{j}+\hat{k}), \\ \hat{\varepsilon}_{i,i+\gamma_{3}}=\frac{1}{\sqrt{6}}(\hat{i}-2\hat{j}+\hat{k}),\text{ }\hat{c}=\frac{1}{\sqrt{3}}(\hat{i}+\hat{j}+\hat{k}) \end{matrix}$ S(23)

and replace with them in S(21), then the Hamiltonian can be expressed as,

$\begin{matrix} H_{i}=J_{1}\sum_{k=1,2,3} \mathbf{S}_{\mathbf{r}_{i}}\cdot\mathbf{S}_{\mathbf{r}_{i}+\mathbf{n}_{k}}+\frac{I}{3}\sum_{k=1,2,3} \mathbf{S}_{\mathbf{r}_{i}}\cdot\mathbf{S}_{\mathbf{r}_{i}+\mathbf{n}_{k}}+\frac{I}{3}(S_{\mathbf{r}_{i}}^{x}\cdot S_{\mathbf{r}_{i}+\mathbf{n}_{1}}^{y}+S_{\mathbf{r}_{i}}^{y}\cdot S_{\mathbf{r}_{i}+\mathbf{n}_{1}}^{x}) \\ \text{ }+\frac{I}{3}(S_{\mathbf{r}_{i}}^{y}\cdot S_{\mathbf{r}_{i}+\mathbf{n}_{2}}^{z}+S_{\mathbf{r}_{i}}^{z}\cdot S_{\mathbf{r}_{i}+\mathbf{n}_{2}}^{y})+\frac{I}{3}(S_{\mathbf{r}_{i}}^{x}\cdot S_{\mathbf{r}_{i}+\mathbf{n}_{3}}^{z}+S_{\mathbf{r}_{i}}^{z}\cdot S_{\mathbf{r}_{i}+\mathbf{n}_{3}}^{x}) \\ \text{ }+\frac{I}{3}(S_{\mathbf{r}_{i}}^{z}\cdot S_{\mathbf{r}_{i}+\mathbf{n}_{1}}^{x}+S_{\mathbf{r}_{i}}^{z}\cdot S_{\mathbf{r}_{i}+\mathbf{n}_{1}}^{y}+S_{\mathbf{r}_{i}}^{x}\cdot S_{\mathbf{r}_{i}+\mathbf{n}_{1}}^{z}+S_{\mathbf{r}_{i}}^{y}\cdot S_{\mathbf{r}_{i}+\mathbf{n}_{1}}^{z}) \\ \text{ }+\frac{I}{3}(S_{\mathbf{r}_{i}}^{x}\cdot S_{\mathbf{r}_{i}+\mathbf{n}_{2}}^{y}+S_{\mathbf{r}_{i}}^{x}\cdot S_{\mathbf{r}_{i}+\mathbf{n}_{2}}^{z}+S_{\mathbf{r}_{i}}^{y}\cdot S_{\mathbf{r}_{i}+\mathbf{n}_{2}}^{x}+S_{\mathbf{r}_{i}}^{z}\cdot S_{\mathbf{r}_{i}+\mathbf{n}_{2}}^{x}) \\ \text{ }+\frac{I}{3}(S_{\mathbf{r}_{i}}^{y}\cdot S_{\mathbf{r}_{i}+\mathbf{n}_{3}}^{z}+S_{\mathbf{r}_{i}}^{y}\cdot S_{\mathbf{r}_{i}+\mathbf{n}_{3}}^{z}+S_{\mathbf{r}_{i}}^{x}\cdot S_{_{\mathbf{r}_{i}+\mathbf{n}_{3}}}^{y}+S_{\mathbf{r}_{i}}^{z}\cdot S_{\mathbf{r}_{i}+\mathbf{n}_{3}}^{y}) \\ \text{ }+\frac{J_{K}}{2}[(S_{\mathbf{r}_{i}}^{x}-S_{\mathbf{r}_{i}}^{y})(S_{\mathbf{r}_{i}+\mathbf{n}_{1}}^{x}-S_{\mathbf{r}_{i}+\mathbf{n}_{1}}^{y})+(S_{\mathbf{r}_{i}}^{y}-S_{\mathbf{r}_{i}}^{z})(S_{\mathbf{r}_{i}+\mathbf{n}_{2}}^{y}-S_{\mathbf{r}_{i}+\mathbf{n}_{2}}^{z})+(S_{\mathbf{r}_{i}}^{z}-S_{\mathbf{r}_{i}}^{x})(S_{\mathbf{r}_{i}+\mathbf{n}_{3}}^{z}-S_{\mathbf{r}_{i}+\mathbf{n}_{3}}^{x})] \\ \text{ }+\frac{J_{\Gamma}+J_{D}}{3\sqrt{2}}(S_{\mathbf{r}_{i}}^{x}+S_{\mathbf{r}_{i}}^{y}-2S_{\mathbf{r}_{i}}^{z})(S_{\mathbf{r}_{i}+\mathbf{n}_{1}}^{x}+S_{\mathbf{r}_{i}+\mathbf{n}_{1}}^{y}+S_{\mathbf{r}_{i}+\mathbf{n}_{1}}^{z})+\frac{J_{\Gamma}-J_{D}}{3\sqrt{2}}[(S_{\mathbf{r}_{i}}^{x}+S_{\mathbf{r}_{i}}^{y}+S_{\mathbf{r}_{i}}^{z})(S_{\mathbf{r}_{i}+\mathbf{n}_{1}}^{x}+S_{\mathbf{r}_{i}+\mathbf{n}_{1}}^{y}-2S_{\mathbf{r}_{i}+\mathbf{n}_{1}}^{z}) \\ \text{ }+\frac{J_{\Gamma}+J_{D}}{3\sqrt{2}}(S_{\mathbf{r}_{i}}^{z}+S_{\mathbf{r}_{i}}^{y}-2S_{\mathbf{r}_{i}}^{x})(S_{\mathbf{r}_{i}+\mathbf{n}_{2}}^{x}+S_{\mathbf{r}_{i}+\mathbf{n}_{2}}^{y}+S_{\mathbf{r}_{i}+\mathbf{n}_{2}}^{z})+\frac{J_{\Gamma}-J_{D}}{3\sqrt{2}}[(S_{\mathbf{r}_{i}}^{x}+S_{\mathbf{r}_{i}}^{y}+S_{\mathbf{r}_{i}}^{z})(S_{\mathbf{r}_{i}+\mathbf{n}_{2}}^{z}+S_{\mathbf{r}_{i}+\mathbf{n}_{2}}^{y}-2S_{\mathbf{r}_{i}+\mathbf{n}_{2}}^{x}) \\ \text{ }+\frac{J_{\Gamma}+J_{D}}{3\sqrt{2}}(S_{\mathbf{r}_{i}}^{z}+S_{\mathbf{r}_{i}}^{x}-2S_{\mathbf{r}_{i}}^{y})(S_{\mathbf{r}_{i}+\mathbf{n}_{3}}^{x}+S_{\mathbf{r}_{i}+\mathbf{n}_{3}}^{y}+S_{\mathbf{r}_{i}+\mathbf{n}_{3}}^{z})+\frac{J_{\Gamma}-J_{D}}{3\sqrt{2}}[(S_{\mathbf{r}_{i}}^{x}+S_{\mathbf{r}_{i}}^{y}+S_{\mathbf{r}_{i}}^{z})(S_{\mathbf{r}_{i}+\mathbf{n}_{3}}^{z}+S_{\mathbf{r}_{i}+\mathbf{n}_{3}}^{x}-2S_{\mathbf{r}_{i}+\mathbf{n}_{3}}^{y}) \\ \text{ }=(J_{1}+\frac{I}{3}+\frac{J_{K}}{2}+\frac{\sqrt{2}J_{\Gamma}}{3}\sum_{k=1,2,3} \mathbf{S}_{\mathbf{r}_{i}}\cdot\mathbf{S}_{\mathbf{r}_{i}+\mathbf{n}_{k}}-(\frac{J_{K}}{2}+\sqrt{2}J_{\Gamma})\sum_{k=1,2,3} S_{\mathbf{r}_{i}}^{\gamma_{k}}S_{\mathbf{r}_{i}+\mathbf{n}_{k}}^{\gamma_{k}} \\ \text{ }+\sum_{k=1,2,3} (\frac{I}{3}+\frac{\sqrt{2}J_{\Gamma}}{3}-\frac{J_{K}}{2})(S_{\mathbf{r}_{i}}^{\alpha_{k}}S_{\mathbf{r}_{i}+\mathbf{n}_{k}}^{\beta_{k}}+S_{\mathbf{r}_{i}}^{\beta_{k}}S_{\mathbf{r}_{i}+\mathbf{n}_{k}}^{\alpha_{k}}) \\ \text{ }+\frac{1}{3}(I-\frac{J_{\Gamma}}{\sqrt{2}})\sum_{k=1,2,3} \sum_{\alpha_{k}\neq\gamma_{k}} (S_{\mathbf{r}_{i}}^{\gamma_{k}}S_{\mathbf{r}_{i}+\mathbf{n}_{k}}^{\alpha_{k}}+S_{\mathbf{r}_{i}}^{\alpha_{k}}S_{\mathbf{r}_{i}+\mathbf{n}_{k}}^{\gamma_{k}}) \\ \text{ }-\frac{J_{D}}{\sqrt{2}}\sum_{k=1,2,3} \sum_{\alpha_{k}\neq\gamma_{k}} (S_{\mathbf{r}_{i}}^{\gamma_{k}}S_{\mathbf{r}_{i}+\mathbf{n}_{k}}^{\alpha_{k}}-S_{\mathbf{r}_{i}}^{\alpha_{k}}S_{\mathbf{r}_{i}+\mathbf{n}_{k}}^{\gamma_{k}}), \end{matrix}$ S(24)

Where *γ_k_* represents *x*, *y*, *z* corresponding to **n**_1_, **n**_2_, **n**_3_ bond, respectively, and *α_k_*, *β_k_* labels the other two components. Hence, there is a relationship between our model parameters and the parameters researchers generally use, that is,

$\begin{matrix} J=J_{1}+\frac{I}{3}+\frac{J_{K}}{2}+\frac{\sqrt{2}J_{\Gamma}}{3}, \\ K=-(\frac{J_{K}}{2}+\sqrt{2}J_{\Gamma}), \\ \Gamma=\frac{I}{3}+\frac{\sqrt{2}J_{\Gamma}}{3}-\frac{J_{K}}{2}, \\ \Gamma'=\frac{1}{3}(I-\frac{J_{\Gamma}}{\sqrt{2}}) \end{matrix}$ S(25)

Reference

[1] H. Liu and G. Khaliullin, Physical Review B **97**, 014407 (2018).

[2] R. Sano, Y. Kato, and Y. Motome, Physical Review B **97**, 014408 (2018).

[3] P. Hofmann, *Solid State Physics: An Introduction* (Wiley-VCH, Deutsche Nationalbibliothek, 2015).

[4] J. Chaloupka, G. Jackeli, and G. Khaliullin, Phys Rev Lett **105**, 027204 (2010).
